# Not All Daydreaming Is Equal: A Longitudinal Investigation of Social and General Daydreaming and Marital Relationship Quality

**DOI:** 10.3389/fpsyg.2022.904025

**Published:** 2022-06-17

**Authors:** Shogo Kajimura, Yuki Nozaki, Takayuki Goto, Jonathan Smallwood

**Affiliations:** ^1^Department of Information and Human Sciences, Kyoto Institute of Technology, Kyoto, Japan; ^2^Faculty of Letters, Konan University, Kobe, Japan; ^3^School of Human Cultures, University of Shiga Prefecture, Shiga, Japan; ^4^Department of Psychology, Queen’s University, Kingston, ON, Canada

**Keywords:** daydreaming, social daydreaming, marital relationship, cross-lagged panel model, attachment style in close relationship

## Abstract

Preliminary evidence suggests that daydreaming about other people has adaptive value in daily social lives. To address this possibility, we examined whether daydreaming plays a role in maintaining close, stable relationships using a 1-year prospective longitudinal study. We found that individuals’ propensity to daydream about their marital partner is separate to general daydreaming. In contrast to general daydreaming, which was associated with lower subsequent relationship investment size (i.e., magnitude and importance of resources attached to a relationship) in the marital partner, partner-related social daydreaming led to a greater subsequent investment size. Additionally, attachment styles moderated these effects. The effect of daydreaming regarding investment size was found only in securely attached individuals. This research advances the emerging field of social daydreaming and highlights self-generated thought as a critical tool that can help people navigate the complex social world.

## Introduction

In daily life, cognition is not always related to events currently occurring. It can arise independently of concurrent perceptual input and any external task being performed. Evidence shows that people spend up to half of their waking time engaged in thoughts that are only loosely tied to their current activity (Kane et al., 2007; Killingsworth and Gilbert, 2010; Song and Wang, 2012). Such self-generated mental activity, often called *daydreaming* (Smallwood, 2013), can help individuals absorbed in it feel happy and calm, and may be used as a coping mechanism to handle the frustrations of daily living (Bigelsen and Schupak, 2011).

Despite these benefits, existing literature suggests that engagement in daydreaming may be detrimental to well-being. For example, daydreaming has been associated with higher levels of anxiety and depression (Stawarczyk et al., 2012), daily unhappiness (Killingsworth and Gilbert, 2010), and poor sleep quality (Carciofo et al., 2014). Furthermore, excessive daydreaming is considered maladaptive daydreaming, which is associated with shame and dissociation (Ferrante et al., 2022), attention deficit hyperactivity disorder, anxiety disorders, depressive disorders, and obsessive-compulsive and related disorders (Somer et al., 2017).

However, daydreaming is not experienced homogenously; consequences of daydreaming may depend on the specific features of imagination patterns (Ruby et al., 2013; Smallwood and Andrews-Hanna, 2013). One important and major content of daydreaming involves other people, which has recently been conceptualized as *social daydreaming* (Poerio and Smallwood, 2016). This study aims to offer novel insights into the emerging field of social daydreaming.

### Social Daydreaming and General Daydreaming

When daydreaming, individuals spend a significant amount of time thinking about others (Mar et al., 2012; Song and Wang, 2012; Andrews-Hanna et al., 2013). One fundamental human motive is the desire to form and maintain social connections (Baumeister and Leary, 1995). Because daydreaming typically reflects engagement with personal goals (Baird et al., 2011), it is associated with the pursuit of social connections (Poerio and Smallwood, 2016). Therefore, it plays an important role regarding social well-being. We define general daydreaming as daydreaming that includes all kinds of content and social daydreaming as daydreaming that only includes social content.

Preliminary evidence suggests that unlike general daydreaming, social daydreaming may have an adaptive value for daily social life. A cross-sectional study revealed that a higher propensity for daydreaming about significant others was related to higher life satisfaction (Mar et al., 2012). A one-day experience sampling study showed that everyday social daydreaming, but not non-social daydreaming, was associated with increased happiness and feelings of connection (Poerio et al., 2015). Additionally, a 4-week longitudinal study demonstrated that positive-valence social daydreaming predicted a reduction in loneliness during the transition to university (Poerio et al., 2016). Given the different functionalities of social daydreaming, these findings highlight the importance of capturing it as a construct separate from general daydreaming.

However, it remains unclear whether social daydreaming has adaptive value within a specific context. Theoretically, daydreaming about another person may facilitate the pursuit and attainment of meaningful social goals, e.g., maintaining a positive relationship with that person (Poerio and Smallwood, 2016). Therefore, it is important to identify who participants daydream about and whether this propensity is connected to the relationship quality with that person. Nevertheless, previous studies did not specify a target of social daydreaming during assessment (Poerio et al., 2016) or capture the relationship quality with a specific other as a correlate of social daydreaming (Mar et al., 2012). Moreover, given that these studies captured the change in adjustments in a maximum of 4 weeks, further long-term research is necessary.

To fill this gap, we focus on a marital partner as a target of social daydreaming and examine how it is associated with marital relationship quality through a 1-year longitudinal study. Marital partners play an important role in an individual’s well-being (Proulx et al., 2007). Therefore, focusing on the propensity for daydreaming of the marital partner as a separate construct from general daydreaming will offer important empirical evidence to deepen the understanding of its interpersonal functionality.

### Attachment Style as a Potential Moderator of the Relationships Between Social Daydreaming and Marital Relationship Quality

Although previous research has generally found positive aspects of social daydreaming, Poerio and Smallwood (2016) stressed that social daydreaming *per se* was not inherently adaptive or maladaptive. Rather, according to the context regulation hypothesis of daydreaming (Smallwood and Andrews-Hanna, 2013), adaptiveness depends on the content of thought (Poerio and Smallwood, 2016). Therefore, a third factor, which could influence the characteristics of daydreams, would moderate the relationship between daydreaming about marital partners and marital relationship quality.

Attachment style is known to influence how people daydream about their marital partners in their daily lives (e.g., Birnbaum et al., 2011). This construct reflects how individuals build mental representations of themselves and significant others based on their interpersonal experiences (Bowlby, 1973; Mikulincer et al., 1993). Although the original attachment theory addresses early relationships between children and caregivers (Bowlby, 1969), it could serve as the basis for the theory of romantic relationships (Hazan and Shaver, 1987).

Among the different attachment styles, individuals with secure attachment to their partner describe love experiences as friendly, happy, and trusting (Hazan and Shaver, 1987). Contrastingly, there are two types of insecure attachment to partners: anxious and avoidant (Hazan and Shaver, 1987; Feeney and Noller, 1990)^1^. Although their detailed features are different,^2^ both types of attachments are characterized by a negatively valanced internal working model (Collins, 1996). Positively and negatively valanced repetitive thoughts are associated with good and poor adjustment, respectively (Segerstrom et al., 2003; Watkins, 2008; Andrews-Hanna et al., 2013).

Recent studies have reported differences in daydreaming experience across attachment styles. Individuals who experienced frequent maladaptive daydreaming tended to show high anxiety, while individuals who experienced normal daydreaming tended to have secure attachment styles (Mariani et al., 2021; Sándor et al., 2021). The relationship between attachment style and problematic social media use was significantly mediated by maladaptive daydreaming in high anxiety-related styles, but not in the secure style (Costanzo et al., 2021). These results indicate that individuals with an insecure attachment style show a greater negative association between daydreaming about their marital partner and subsequent marital relationship quality, whereas individuals with a secure attachment style show a greater positive association between them.

### The Present Study

We examined how the propensity for daydreaming of a marital partner was associated with marital relationship quality over 1 year, *via* a three-wave longitudinal study. Following an emerging body of research on social daydreaming, we conceptualized the propensity for daydreaming about the marital partner as a separate construct from the propensity for general daydreaming. We modified an existing daydreaming measure by emphasizing that the target was an individual’s marital partner. We expected to find two separate but related factors when we jointly analyzed the existing measure of general daydreaming and modified measures of social daydreaming (Hypothesis 1).

Moreover, given that social daydreaming generally has adaptive value for daily social lives, we hypothesized that the daydreaming of marital partners would be positively associated with subsequent marital relationship quality (Hypothesis 2). We also hypothesized that attachment style would moderate this association; individuals with a secure attachment style would show a greater positive association between daydreaming about their marital partner and subsequent marital relationship quality, whereas individuals with an insecure attachment style would show a greater negative association between them. (Hypothesis 3). Because gender and marital duration are related to marital relationship quality (Proulx et al., 2007), we used them as control variables.

## Materials and Methods

### Participants and Procedure

All participants were Japanese and married, and were recruited by an online survey company (Rakuten Insight, Japan).^3^ The survey was conducted in Japanese. The data supporting the findings of this study are available upon request from the corresponding author. The surveys were conducted in January 2016 (Time 1), July 2016 (Time 2), and January 2017 (Time 3). To increase the quality of the data, the online survey company screened participants who failed to correctly answer at least one of the two screening questions (“Please select the leftmost option” and “Please select the rightmost option”) at Time 1. Thus, we obtained data only from participants who answered these questions correctly.

At Time 1, 327 participants (156 men and 171 women; mean age = 42.6, *SD* = 12.80, age range = 22–69 years) completed the study. At Times 2 and 3, 251 participants (119 men and 132 women) and 215 participants (102 men and 113 women), respectively, completed all questionnaires. Participants provided personal information (marital duration, family composition, occupation, salary, and educational background) at Time 1. At each time point, the participants completed a set of questionnaires, as detailed below. They also completed several individual difference measures unrelated to the current research question (see Supplementary Material 1 for a full description of these measures). The study procedure was approved by the ethics committee of a university in Japan.

The sample size was determined *a priori* by considering similar prior studies and budget constraints. The required sample size to detect misfit was satisfied, corresponding to root mean square error of approximation (RMSEA) = 0.08, involving *df* > 15, with a power of 95% on α = 0.05. This power analysis was followed by Moshagen and Erdfelder (2015) procedure and conducted using the semPower 1.1.0 package (Moshagen, 2020).

### Measures

#### Propensity for General and Partner-Related Daydreaming (Time 1–3)

General daydreaming was assessed using the Daydream Frequency Scale (DDFS; Giambra, 1993), which composes the Imaginal Process Inventory (Singer and Antrobus, 1970). It consists of 12 items and measures the frequency of general daydreams in daily life. Previous research has validated the Japanese version of the DDFS (Kajimura and Nomura, 2016). In addition, we slightly modified the DDFS to measure the propensity for partner-related daydreaming (DDFS-partner) by simply adding the phrase “about your partner” after the word “daydreaming” in the instruction and items of the DDFS. Both scales were answered on a 5-point scale.

#### Marital Relationship Quality (Time 1–3)

The subjective quality of marital relationships was measured using the Investment Model Scale (IMS; Rusbult et al., 1998). It measures commitment (intent to persist in a relationship; *via* seven items), satisfaction (positive versus negative affect experienced in a relationship; five items), investment size (magnitude and importance of resources attached to a relationship; five items), and quality of alternative relationships (perceived desirability of the best available alternative to a relationship; five items), which enabled us to explore the effect of daydreaming on various factors associated with marital relationship quality. The items were answered on a 7-point scale (1 = *strongly disagree* to 7 = *strongly agree*). The Japanese version of the IMS was validated in a previous study (Komura and Nakamine, 2013).

#### Attachment Style (Time 1)

Attachment style was measured using the Experiences in Close Relationship Scale-Short Form (ECR-S; Wei et al., 2007), which consists of 12 items answered on a 7-point scale (1 = *strongly disagree* to 7 = *strongly agree*). It measures two attachment dimensions: anxiety (fear of interpersonal rejection or abandonment, excessive need for approval from others, and distress when one’s partner is unavailable or unresponsive), and avoidance (fear of dependence and interpersonal intimacy, excessive need for self-reliance, and reluctance to self-disclose). The original items were translated into Japanese by the authors, and consistency was checked *via* back-translation (NAI, Inc., Japan).^4^

### Statistical Analysis

All analyses were conducted with R 4.0.3 (R Core Team, 2020) and Mplus 8.3 (Muthén and Muthén, 1998–2017) *via* the MplusAutomation 0.8 package (Hallquist and Wiley, 2018). As in most longitudinal studies, data resulting from participant attrition were missing. Thus, we compared participants who remained in the study and those who dropped out using Little’s Missing Completely at Random Test (Little, 1988) with the BaylorEdPsych 0.5 package (Beaujean, 2012). We obtained a non-significant χ^2^ value for this test (χ^2^(42) = 47.79, *p* = 0.249), suggesting that missing values in the dataset were completely missing at random and could be reliably estimated. Therefore, we applied the full information maximum likelihood method to structural equation modeling to address missing data (Enders, 2010).

#### Factor Analyses of Daydreaming Scales

To investigate whether partner-related and general daydreaming can be regarded as separate (but related) factors, we first conducted cross-sectional factor analyses on the data from each time point separately. Following a recent recommendation for factor analyses (Schmitt et al., 2018), we first conducted a parallel analysis using minimum rank factor analysis (Timmerman and Lorenzo-Seva, 2011) with the EFA.MRFA 1.0.9 package (Navarro-Gonzalez and Lorenzo-Seva, 2020) to initially assess the number of factors. We then conducted exploratory factor analyses (EFA) using promax rotation with the psych 2.0.9 package (Revelle, 2020).

#### Longitudinal Measurement Invariance

We examined the longitudinal measurement invariance for daydreaming and investment scales. We tested a series of four models with increasing invariance for each subscale (Widaman et al., 2010): (1) *configural invariance* (same pattern of fixed and free factor loadings across time), (2) weak factorial invariance (invariant factor loadings across time), (3) strong factorial invariance (invariant factor loadings and intercepts across time), and (4) strict factorial invariance (invariant factor loadings, intercepts, and unique variances across time). To identify the same latent construct longitudinally, a strong or strict factorial invariance must hold across measurement times (Widaman et al., 2010, p.13).

To evaluate the invariance between successive models, we investigated the changes in the comparative fit index (ΔCFI) because the chi-square difference test is sensitive to large samples (Cheung and Rensvold, 2002). We followed the rule of thumb of Cheung and Rensvold (2002) that a CFI decrease ≤ 0.01 indicates an invariant model fit. All models were estimated using robust full information maximum likelihood to accommodate non-normality. Residual correlations between identical items over time were freely estimated to account for the non-independence of uniqueness over time.

#### Descriptive Statistics

We report the means and standard deviations of each variable. Moreover, we evaluated internal consistency using McDonald’s (1999) total omega coefficient (ω_*t*_) with the psych 2.0.9 package (Revelle, 2020). This coefficient is superior to Cronbach’s alpha in evaluating a scale’s internal consistency (Revelle and Zinbarg, 2009; McNeish, 2018).

#### Cross-Lagged Panel Models

**FIGURE 1:**
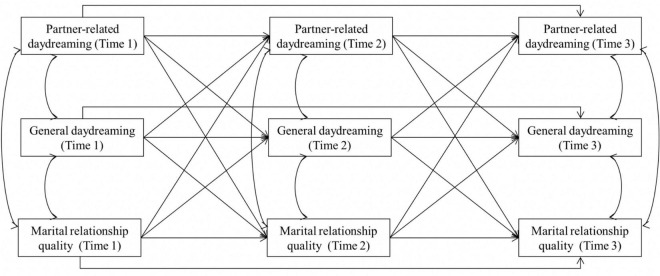
Cross-lagged model. Control variables (gender and marital duration) are not displayed for clarity of presentation. Marital relationship quality is indicated by any subscale of the Investment Model Scale.

Our main interest was between-person effects (i.e., when individuals have a high propensity for partner-related daydreaming relative to others, they experience a subsequent rank-order increase in marital relationship quality versus individuals with a low propensity for partner-related daydreaming) rather than within-person effects (i.e., when individuals have a higher propensity for partner-related daydreaming than usual, they experience a subsequent increase in marital relationship quality). Therefore, we analyzed the longitudinal relationships between daydreaming and marital relationship quality using cross-lagged panel models (Figure 1), following the recommendations of Orth et al. (2021).

We tested bidirectional relationships among observed partner-related daydreaming, general daydreaming, and marital relationship quality at three time points. We also included first-order autoregressive paths (i.e., stability paths from Time 1 to Time 2 and from Time 2 to Time 3 for all variables), second-order autoregressive paths (i.e., stability paths from Time 1 to Time 3 for all variables), and within-time correlations among all the variables. The corresponding path coefficients were constrained to be equal across intervals, and gender and marital duration were set as the control variables. To evaluate the overall model fit, we used the following indices (Browne and Cudeck, 1992; Hu and Bentler, 1999): CFI ≥ 0.90, standardized root mean square residual (SRMR) ≤ 0.08, and RMSEA ≤ 0.08.

### Specifying Different Type of Attachment Style

Following previous research (Kawamoto, 2016), we attempted to specify different types of attachment styles based on anxiety and avoidance scores. Thus, we conducted a two-stage cluster analysis, following the procedure recommended by Punj and Stewart (1983). This procedure has frequently been used in psychological research (Luyckx et al., 2005; Stellar et al., 2020). All scores were standardized prior to analysis. First, we carried out a hierarchical cluster analysis using Ward’s method on squared Euclidean distances to identify the number of clusters. We examined the dendrogram and clustering validity indices from the NbClust package (version 3.0; Charrad et al., 2014). We then conducted *k*-means non-hierarchical cluster analysis using the number of clusters and centroids identified using Ward’s method to form the final clusters.

### Multigroup Cross-Lagged Panel Models

To test whether attachment style moderated the cross-lagged effects, we conducted a multigroup cross-lagged analysis. We tested whether model fit was significantly worsened by equality constraints across the groups for cross-lagged effects of social daydreaming and general daydreaming on subsequent marital relationship quality. If the chi-square difference test revealed that model fit was significantly worsened by the equality constraint, the cross-lagged effects were moderated by attachment style.

## Results

### Factor Analyses for Daydreaming Scales

Parallel analysis using minimum rank factor analysis recommended two common factors for general daydreaming and partner-related items at all the time points. Thus, we conducted an EFA by setting two factors in the data from each time point separately. Table 1 reports the factor loadings and correlations obtained from the EFA. At all time points, we found two positively correlated factors that clearly corresponded to partner-related and general daydreaming. All items loaded highly on the intended factor, with coefficients exceeding 0.40. Moreover, none of the items exhibited cross-factor loadings exceeding an absolute value of 0.27. These results support Hypothesis 1.

**TABLE 1:** Factor loadings and correlations based on the exploratory factor analyses of the general and partner-related daydreaming scale items.

	Time 1		Time 2		Time 3	
Item	Factor 1	Factor 2	Factor 1	Factor 2	Factor 1	Factor 2
DDFS 01	−0.14	**0.94**	−0.11	**0.90**	−0.18	**0.96**
DDFS 02	−0.03	**0.69**	−0.11	**0.72**	0.03	**0.64**
DDFS 03	0.00	**0.76**	0.13	**0.57**	0.27	**0.50**
DDFS 04	−0.14	**0.85**	0.04	**0.63**	0.17	**0.57**
DDFS 05	−0.01	**0.84**	0.00	**0.85**	−0.07	**0.91**
DDFS 06	0.00	**0.66**	0.07	**0.64**	−0.01	**0.72**
DDFS 07	0.15	**0.64**	0.18	**0.65**	0.05	**0.71**
DDFS 08	0.26	**0.42**	0.04	**0.64**	0.20	**0.54**
DDFS 09	0.22	**0.39**	−0.02	**0.75**	0.20	**0.57**
DDFS 10	0.26	**0.48**	0.08	**0.66**	0.19	**0.56**
DDFS 11	0.02	**0.81**	−0.04	**0.81**	−0.10	**0.89**
DDFS 12	0.01	**0.75**	−0.01	**0.67**	−0.04	**0.73**
DDFS-P 01	**0.74**	0.09	**0.78**	0.01	**0.85**	−0.04
DDFS-P 02	**0.46**	0.15	**0.56**	0.08	**0.68**	−0.01
DDFS-P 03	**0.65**	0.14	**0.76**	−0.05	**0.81**	−0.08
DDFS-P 04	**0.59**	0.15	**0.66**	0.07	**0.76**	−0.01
DDFS-P 05	**0.83**	0.00	**0.88**	−0.01	**0.78**	0.07
DDFS-P 06	**0.65**	0.07	**0.71**	0.05	**0.52**	0.23
DDFS-P 07	**0.81**	0.00	**0.85**	−0.01	**0.73**	0.07
DDFS-P 08	**0.93**	−0.16	**0.81**	−0.08	**0.83**	−0.04
DDFS-P 09	**0.83**	−0.11	**0.72**	0.05	**0.80**	−0.03
DDFS-P 10	**0.85**	−0.11	**0.77**	0.00	**0.78**	0.00
DDFS-P 11	**0.77**	0.07	**0.76**	−0.02	**0.67**	0.11
DDFS-P 12	**0.81**	−0.02	**0.76**	0.02	**0.80**	0.02
Factor correlation	0.69		0.61		0.71	

*DDFS = the daydream frequency scale; DDFS-P = the daydream frequency scale for partner. Factor loadings ≥ 0.30 are in boldface.*

### Longitudinal Measurement Invariance

We examined the longitudinal measurement invariance of partner-related daydreaming, general daydreaming, and each subscale of marital relationship quality. Table 2 reports the results of the model fit indices and the nested model comparisons. Strict factorial invariances were achieved for all variables (CFI decrease ≤ 0.01),^5^ except for the alternatives. Therefore, longitudinal relationships associated with alternatives should be interpreted with caution.

**TABLE 2:** Model-fit indices and nested model comparisons from the analyses of longitudinal measurement invariance.

Nested invariance model	SB-χ^2^	*df*	SRMR	RMSEA [90% CI]	CFI	Model comparison	ΔCFI
**Partner-related daydreaming**							
A. Configural invariance	1676.04***	555	0.065	0.079 [0.074, 0.083]	0.814	–	–
B. Weak factorial invariance	1683.62***	579	0.074	0.076 [0.072, 0.081]	0.817	B vs. A	0.003
C. Strong factorial invariance	1758.11***	603	0.073	0.077 [0.072, 0.081]	0.809	C vs. B	−0.008
D. Strict factorial invariance	1778.04***	627	0.077	0.075 [0.071, 0.079]	0.809	D vs. C	0.000
**General daydreaming**							
A. Configural invariance	1292.03***	555	0.061	0.064 [0.059, 0.068]	0.863	–	–
B. Weak factorial invariance	1321.39***	579	0.066	0.063 [0.058, 0.067]	0.862	B vs. A	−0.001
C. Strong factorial invariance	1399.05***	603	0.069	0.064 [0.059, 0.068]	0.852	C vs. B	−0.010
D. Strict factorial invariance	1415.86***	627	0.070	0.062 [0.058, 0.066]	0.854	D vs. C	0.002
**Commitment**							
A. Configural invariance	427.10***	165	0.044	0.070 [0.062, 0.078]	0.925	–	–
B. Weak factorial invariance	443.74***	179	0.058	0.067 [0.059, 0.075]	0.925	B vs. A	0.000
C. Strong factorial invariance	486.11***	193	0.064	0.068 [0.061, 0.076]	0.917	C vs. B	−0.008
D. Strict factorial invariance	479.69***	207	0.068	0.063 [0.056, 0.071]	0.922	D vs. C	0.005
**Satisfaction**							
A. Configural invariance	150.22***	72	0.027	0.058 [0.045, 0.071]	0.971	–	–
B. Weak factorial invariance	166.96***	82	0.052	0.056 [0.044, 0.069]	0.969	B vs. A	−0.002
C. Strong factorial invariance	196.55***	92	0.050	0.059 [0.048, 0.070]	0.962	C vs. B	−0.007
D. Strict factorial invariance	231.30***	102	0.057	0.062 [0.052, 0.073]	0.953	D vs. C	−0.009
**Investment**							
A. Configural invariance	125.07***	72	0.048	0.047 [0.033, 0.061]	0.962	–	–
B. Weak factorial invariance	136.74***	82	0.057	0.045 [0.031, 0.058]	0.961	B vs. A	−0.001
C. Strong factorial invariance	152.12***	92	0.059	0.045 [0.032, 0.057]	0.957	C vs. B	−0.004
D. Strict factorial invariance	165.98***	102	0.066	0.044 [0.031, 0.056]	0.955	D vs. C	−0.002
**Alternatives**							
A. Configural invariance	79.28	72	0.050	0.018 [0.000, 0.038]	0.992	–	–
B. Weak factorial invariance	99.06	82	0.063	0.025 [0.000, 0.042]	0.981	B vs. A	−0.011
C. Strong factorial invariance	110.20	92	0.062	0.025 [0.000, 0.042]	0.979	C vs. B	−0.002
D. Strict factorial invariance	151.43**	102	0.082	0.038 [0.025, 0.051]	0.944	D vs. C	−0.035

*SB-χ^2^ = Satorra-Bentler scaled chi-square; df = degrees of freedom; SRMR = standardized root mean squared residual; RMSEA = root mean squared error of approximation; CI = confidence interval; CFI = comparative fit index.*

**p < 0.01, ***p < 0.001.

### Descriptive Statistics

Table 3 summarizes the means, standard deviations, and McDonald’s (1999) total omega coefficients (ω_*t*_) for each variable. Although the internal consistency was slightly low for anxiety, it was satisfactory for the remaining variables.

**TABLE 3:** Mean, standard deviation, and total omega coefficient of each variable.

	Time 1		Time 2		Time 3	
Variable	*M* (*SD*)	ω_*t*_	*M* (*SD*)	ω_*t*_	*M* (*SD*)	ω_*t*_
Demographics						
Gender*^a^*	0.52 (0.50)	–	–		–	
Marital duration	15.37 (12.79)	–	–		–	
Attachment						
Anxiety	3.48 (0.86)	0.63	–		–	
Avoidance	3.19 (1.06)	0.80	–		–	
Daydreaming						
Partner-related daydreaming	1.81 (0.69)	0.94	1.62 (0.60)	0.94	1.67 (0.62)	0.95
General daydreaming	2.14 (0.76)	0.93	1.99 (0.73)	0.93	1.98 (0.74)	0.93
Relationship quality						
Commitment	5.54 (1.29)	0.92	5.28 (1.29)	0.92	5.31 (1.23)	0.92
Satisfaction	4.97 (1.50)	0.95	4.79 (1.45)	0.95	4.75 (1.40)	0.95
Investment	4.14 (1.23)	0.83	4.00 (1.21)	0.85	3.98 (1.16)	0.84
Alternatives	3.74 (1.06)	0.75	3.74 (1.02)	0.77	3.68 (1.01)	0.79

*^a^Male = 0, Female = 1.*

### Cross-Lagged Panel Models

We analyzed the bidirectional longitudinal relationships between partner-related daydreaming, general daydreaming, and each subscale of marital relationship quality using cross-lagged panel models (Figure 1). The model fits were adequate for all models (CFIs > 0.981, SRMRs < 0.049, RMSEAs < 0.063). The results showed that all cross-lagged effects were non-significant, except for investment (see Supplementary Material 2 for full parameter estimates).

Table 4 summarizes the cross-lagged effects of the relationships between social daydreaming, general daydreaming, and investment. Specifically, the cross-lagged effect of partner-related daydreaming on investment is positive and statistically significant (β = 0.18, *p* = 0.001). Contrastingly, the cross-lagged effect of general daydreaming on investment is negative and statistically significant (β = −0.13, *p* = 0.008). The remaining cross-lagged effects were not statistically significant. These results suggest that investment supports Hypothesis 2.

**TABLE 4:** Unstandardized and standardized coefficients of the cross-lagged effects.

	*B* [95% CI]	β
Partner-related daydreaming → Investment	0.33** [0.14, 0.52]	0.18
General daydreaming → Investment	−0.21** [−0.37, −0.05]	−0.13
Investment → Partner-related daydreaming	0.02 [−0.02, 0.06]	0.04
Investment → General daydreaming	0.01 [−0.03, 0.06]	0.02
Partner-related daydreaming → General daydreaming	0.05 [−0.08, 0.18]	0.05
General daydreaming → Partner-related daydreaming	0.04 [−0.04, 0.12]	0.05

*B = unstandardized coefficient. CI, confidence interval. β = average standardized coefficient.*

**p < 0.01.

### Moderated Effects of the Attachment Style

Cluster analysis revealed three attachment styles that corresponded to previous studies (Hazan and Shaver, 1987; Kawamoto, 2016): secure (low anxiety and low avoidance; *n* = 118), anxious (high anxiety and middle avoidance; *n* = 76), and avoidant (middle anxiety and high avoidance; *n* = 133). We report the details of the cluster analysis and differences in descriptive values across attachment styles in Supplementary Material 3.

Next, we conducted multigroup analyses to investigate whether attachment style moderated the cross-lagged effects. The chi-square difference tests revealed that this equality constraint significantly worsened the model fit for investment (Δχ^2^(4) = 14.76, *p* = 0.005) but not for commitment (Δχ^2^(4) = 1.36, *p* = 0.850), satisfaction (Δχ^2^(4) = 6.59, *p* = 0.159), and alternatives (Δχ^2^(4) = 5.43, *p* = 0.246). These results suggest that attachment style moderates the cross-lagged effects of social daydreaming and general daydreaming on investment.^6^

Table 5 reports cross-lagged effects from social daydreaming and general daydreaming to investment per attachment style from the model without equality constraint. The model fit was adequate (χ^2^(45) = 57.23, *p* = 0.105, CFI = 0.988, SRMR = 0.052, RMSEA = 0.050 90% CI [0.000, 0.085]). Specifically, in the secure group, the cross-lagged effect of partner-related daydreaming on investment was positive and statistically significant (β = 0.43, *p* < 0.001), whereas the cross-lagged effect of general daydreaming on investment was negative and statistically significant (β = −0.34, *p* < 0.001). These cross-lagged effects were negligible and non-significant in the anxious and avoidant groups. These results partially supported Hypothesis 3.

**TABLE 5:** Cross-lagged effects from social daydreaming and general daydreaming to investment per attachment style.

	Secure (*n* = 118)		Anxious (*n* = 76)		Avoidant (*n* = 133)	
	*B* [95% CI]	β	*B* [95% CI]	β	*B* [95% CI]	β
Partner-related daydreaming → Investment	0.66*** [0.39, 0.94]	0.43	0.16 [−0.11, 0.43]	0.10	0.06 [−0.23, 0.34]	0.03
General daydreaming → Investment	−0.48*** [−0.72, −0.23]	−0.34	−0.02 [−0.30, 0.25]	−0.02	0.01 [−0.20, 0.21]	0.00

*B = unstandardized coefficient. CI, confidence interval. β = average standardized coefficient.*

***p < 0.001.

## Discussion

### Summary of Results

Our study provides important evidence that the social features of daydreams can play an important role in marital relationship quality, especially for the investments people make in their marital relationships. Consistent with Hypothesis 1, we found that individuals’ propensity to daydream about their marital partners was a separate factor from general daydreaming. Moreover, we found that partner-related daydreaming and general daydreaming had opposing effects on investment in marital partners. In contrast to general daydreaming, which had a negative impact on subsequent investment size in the marital partner, thinking about the marital partner at idle moments led to larger subsequent investments. These results are consistent with Hypothesis 2. Additionally, attachment styles moderated these cross-lagged effects. We found a positive effect of partner-related daydreaming and a negative effect of general daydreaming with investment only in the secure group, partially supporting Hypothesis 3.

### Theoretical Implications

Our study contributes to the theoretical advancement of social daydreaming (Poerio and Smallwood, 2016). A growing body of evidence has demonstrated that understanding the consequences of daydreaming in daily life requires an understanding of the specific features of the pattern of thought (Smallwood and Andrews-Hanna, 2013). Our research extends these findings by showing that in the context of close personal relationships, the daydreaming of a marital partner is functionally distinct from general daydreaming. Hence, our results are theoretically important because they provide empirical evidence showing the uniquely positive consequences of social daydreaming in the context of marital relationships.

We find these effects only for investment size in marital partners, possible since only the investment size has a property—accumulation, which is directly affected by the propensity of partner-related daydreaming. Daydreaming about a partner can be considered a way in which people put time and effort into their relationship, and prior studies suggest that the more often an individual spends time thinking about the partner, the more resources are accumulated in the relationship (Rusbult, 1983; Rusbult et al., 1998; Le and Agnew, 2003). This directed relationship is reflected in the results of the cross-lagged models; the direction from partner-related daydreaming to investment size, but not from investment size to partner-related daydreaming, was significant. Contrastingly, other subscales of relationship quality are unlikely to be directly affected by partner-related daydreaming. Satisfaction level is influenced by the extent to which the partner fulfills an individual’s most important needs, indicating that the partner’s propensity to daydream about the individual is more important for the individual’s satisfaction level. The quality of the alternatives is based on the situation “outside” the current relationship, which is independent of the propensity to daydream about the partner. Commitment emerges because of the investment size, satisfaction level, and quality of the alternatives; thus, the effect of partner-related daydreaming on commitment would be blurred.

Our study also provides further insight into the context regulation hypothesis of daydreaming (Smallwood and Andrews-Hanna, 2013; Smallwood and Schooler, 2015). Consistent with this hypothesis, the results clearly highlight the fact that the benefits of social daydreaming are not universal. Specifically, individuals with anxious and avoidant attachment styles did not show a significant association between partner-related daydreaming and subsequent investment in their partner. This might be because daydreaming about marital partners was only a way of compensation for individuals with insecure attachment styles. Individuals who display frequent maladaptive daydreaming, which is associated with insecure attachment styles (Costanzo et al., 2021; Mariani et al., 2021; Sándor et al., 2021), tend to address unmet emotional needs by engaging in compensatory fantasies specific to personality traits (Brenner et al., 2022). If partner-related daydreaming served the sole purpose of compensation in individuals with insecure attachment styles, it would be reasonable that the time spent daydreaming did not reflect in the relationship. The hypothetical compensatory function of daydreaming in individuals with insecure attachment styles should be tested in future research.

Contrastingly, individuals with a secure attachment style showed a positive association between partner-related daydreaming and subsequent investment in their marital partners. This result is reasonable, as secure individuals have a positively valenced model of the social world (Collins, 1996), and positively valenced repetitive thought is associated with positive adjustment (Segerstrom et al., 2003; Watkins, 2008; Andrews-Hanna et al., 2013). Thus, positively valenced daydreaming about their partners, which is daydreaming not for compensation or other purposes but for the sake of daydreaming itself, could reflect positively in relationships.

### Limitations and Future Directions

Despite the strengths of this study, it has certain limitations. First, it would have been helpful to use online experience sampling in daily life to examine the situations in which individuals use self-generated thoughts to facilitate smooth social relationships. While our study reveals the long-term effect of socially focused daydreaming on social relationships, momentary experience sampling allows for the assessment of short-term effects of ongoing thoughts on subsequent behaviors. This fills the gap between thinking about another person on the one hand, and the pursuit and attainment of meaningful social goals on the other (Poerio and Smallwood, 2016).

Second, investigating the mutual effect of thinking about a partner in marital relationships will further expand this study’s findings. Although this study reveals that individuals’ partner-related daydreaming affects their subsequent subjective investment size, it is possible that this association would have maximum benefits for relationships in which both partners engage in these patterns of cognition.

Third, we identified three attachment styles from the empirical data, in spite of the presence of existing studies using four attachment styles (e.g., Brennan et al., 1998). Specifically, the collected data were best fitted to the model omitting the cluster of fearful attachment style, which represents both high anxiety and avoidance (Brennan et al., 1998). This is consistent with some previous studies, including a study with Japanese participants, which identified the same three clusters from empirical data (e.g., Ceglian and Gardner, 2001; Kawamoto, 2016). Moreover, as this study mainly focused on the difference between secure and insecure styles, the difference in the number of clusters did not remarkably affect hypotheses testing. Nevertheless, future research should examine whether the fearful attachment style can be identified with larger samples.

Fourth, the sample we studied was only from Japan. It is possible that important cultural boundaries in our results will determine for whom and when socially focused daydreaming is most likely to have adaptive functions. However, we would like to emphasize that our results are consistent with the existing theory, which is largely based on Western population studies (Smallwood and Andrews-Hanna, 2013; Poerio and Smallwood, 2016).

## Conclusion

Our study showed that social daydreaming plays an important role in marital relationships. Moreover, daydreaming about a marital partner has beneficial associations with investment in the partner over a relatively long period of time. Given the important role of social relationships, our study highlights that periods of self-generated thinking are not always idle fantasies but can play a crucial role as one of the most defining features of the human condition.

## Data Availability Statement

The raw data supporting the conclusions of this article will be made available by the authors, without undue reservation.

## Ethics Statement

The studies involving human participants were reviewed and approved by the Ethical Committees of the Kyoto University. The patients/participants provided their written informed consent to participate in this study.

## Author Contributions

SK: conceptualization, data collection, and writing the manuscript. YN: conceptualization, statistical analysis, and writing the manuscript. TG: conceptualization and statistical analysis. JS: supervision and editing the manuscript. All authors contributed to the article and approved the submitted version.

## Conflict of Interest

The authors declare that the research was conducted in the absence of any commercial or financial relationships that could be construed as a potential conflict of interest.

## Publisher’s Note

All claims expressed in this article are solely those of the authors and do not necessarily represent those of their affiliated organizations, or those of the publisher, the editors and the reviewers. Any product that may be evaluated in this article, or claim that may be made by its manufacturer, is not guaranteed or endorsed by the publisher.

## Footnotes

1 Although existing literature using four attachment styles is prevalent (e.g., Brennan et al., 1998), some previous studies have identified three clusters consisting of secure, anxious, and avoidant styles from empirical data (e.g., Ceglian and Gardner, 2001; Kawamoto, 2016). Considering this inconsistency, we explored the number of attachment styles based on the empirical data, following the statistical procedure used in a study by Brennan et al. (1998) detailed in the Method section. Three styles were identified from the current data and used in this study, as indicated by the results (Supplementary Figures 1, 2). This point will be discussed in the section “Discussion.”

2 Anxious attachment is linked to a fear of interpersonal rejection, excessive need for approval from others, and distress when one’s partner is unavailable or unresponsive, whereas avoidant attachment is linked to a fear of dependence and interpersonal intimacy, excessive need for self-reliance, and reluctance to self-disclose (Ainsworth et al., 1978; Hazan and Shaver, 1987).

3
https://research.rakuten.co.jp/en/aboutus.html

4
https://www.nai.co.jp/

5 The absolute values of CFI were not satisfactory for partner-related daydreaming and general daydreaming. However, Moshagen and Auerswald (2018) strongly recommend not using the absolute value of CFI in evaluating the fit of a factor analysis model, despite being useful for model comparison. This is because CFI reflects not only amount of discrepancy but also loading magnitude (i.e., lack of indicator reliability). For this reason, “…a superior approach is to consider indices that are pure indicators of model fit or pure indicators of construct reliability (Moshagen et al., 2020, p.187).” Thus, we did not consider the absolute value of CFI for evaluating each factor model.

6 Although we did not have a specific hypothesis, we tested whether model fit was significantly worsened by equality constraint for cross-lagged effects from marital relationship quality to social daydreaming and general daydreaming across attachment styles. The chi-square difference tests revealed that this equality constraint significantly worsen model fit for alternative (Δχ^2^(4) = 19.90, *p* < 0.001), whereas not for commitment (Δχ^2^(4) = 1.47, *p* = 0.832), satisfaction (Δχ^2^(4) = 1.86, *p* = 0.762), and investment (Δχ^2^(4) = 2.49, *p* = 0.647). They suggest that attachment style moderated cross-lagged effects from alternative to social daydreaming and general daydreaming. Specifically, the cross-lagged effect of alternative on partner-related daydreaming was negative and statistically significant in the secure group (β = 0.18, *p* = 0.001). However, it was negligible and non-significant in the anxious (β = 0.07, *p* = 0.369) and avoidant groups (β = −0.03, *p* = 0.557). The cross-lagged effect of alternative on general daydreaming was non-significant in all groups. The model fit was adequate (χ^2^(45) = 54.94, *p* = 0.147, CFI = 0.990, SRMR = 0.049, RMSEA = 0.045 90% CI [0.000, 0.082]).
