# Supplementary material for: Not All Daydreaming Is Equal: A Longitudinal Investigation of Social and General Daydreaming and Marital Relationship Quality
Source: Front Psychol. 2022 Jun 17;13:904025. doi: 10.3389/fpsyg.2022.904025 (PMC9247565; doi:10.3389/fpsyg.2022.904025)
Supplement: Supplementary file 1 [file Data_Sheet_1.docx]

**Supplemental Materials**

**Not All Daydreaming is Equal: A Longitudinal Investigation of Social and General Daydreaming and Marital Relationship Quality**

**Supplemental Material 1: Additional individual difference measures.**

The questionnaire includes additional scales for other research purposes. At Times 1–3, participants answered the Gratitude Questionnaire-6 (McCullough et al., 2002), the Appreciation in Relationships Scale (Gordon et al., 2012), and an original scale for measuring the subjective feeling about how well one understands your partner’s feelings and how similar they are to their partner’s. Moreover, participants also answered the Big Five Inventory (John et al., 1991), the revised Life Orientation Test (Scheier et al., 1994), and a brief version of the Need for Closure Scale (Roets & Van Hiel, 2011) at Time 1 only.

**Supplemental Material 2: Parameter estimates from the cross-lagged models.**

| Commitment (Model fit: χ^2^(15) = 31.45, *p* = .008, CFI = .985, SRMR = .049, RMSEA = .058 90% CI [.029, .086]) | | | | | | |
| --- | --- | --- | --- | --- | --- | --- |
| Cross-lagged effects | *B* [95% CI] | β |  |  |  |  |
| Partner-related daydreaming → Commitment | 0.06 [-0.10, 0.21] | .03 |  |  |  |  |
| General daydreaming → Commitment | -0.01 [-0.12, 0.11] | .00 |  |  |  |  |
| Commitment → Partner-related daydreaming | 0.02 [-0.01, 0.06] | .05 |  |  |  |  |
| Commitment → General daydreaming | 0.02 [-0.02, 0.05] | .03 |  |  |  |  |
| Partner-related daydreaming → General daydreaming | 0.05 [-0.07, 0.18] | .05 |  |  |  |  |
| General daydreaming → Partner-related daydreaming | 0.05 [-0.03, 0.13] | .06 |  |  |  |  |
| First-order autoregressive effects |  |  |  |  |  |  |
| Partner-related daydreaming | 0.45^***^ [0.33, 0.57] | .48 |  |  |  |  |
| General daydreaming | 0.53^***^ [ 0.42, 0.64] | .54 |  |  |  |  |
| Commitment | 0.70^***^ [ 0.62, 0.78] | .71 |  |  |  |  |
| Second-order autoregressive effects |  |  |  |  |  |  |
| Partner-related daydreaming | 0.22^***^ [ 0.12, 0.33] | .26 |  |  |  |  |
| General daydreaming | 0.24^***^ [ 0.12, 0.37] | .25 |  |  |  |  |
| Commitment | 0.16^*^ [ 0.03, 0.28] | .16 |  |  |  |  |
| Concurrent correlations | Time 1  *r* [95% CI] |  |  | Time 2  *r* [95% CI] |  | Time 3  *r* [95% CI] |
| Partner-related daydreaming ↔ Commitment | .15^**^ [ .04, .26] |  |  | .10 [-.03, .23] |  | .12 [-.03, .26] |
| General daydreaming ↔ Commitment | .01 [-.10, .12] |  |  | .01 [-.17, .15] |  | .00 [-.14, .14] |
| Partner-related daydreaming ↔ General daydreaming | .68^***^ [.59, .76] |  |  | .55^***^ [ .41, .69] |  | .65^***^ [ .54, .75] |
| Satisfaction (Model fit: χ^2^(15) = 34.54, *p* = .003, CFI = .981, SRMR = .048, RMSEA = .063 90% CI [.035, .091]) | | | | | | |
| Cross-lagged effects | *B* [95% CI] | β |  |  |  |  |
| Partner-related daydreaming → Satisfaction | 0.14 [-0.02, 0.30] | .07 |  |  |  |  |
| General daydreaming → Satisfaction | 0.00 [-0.13, 0.13] | .00 |  |  |  |  |
| Satisfaction → Partner-related daydreaming | 0.02 [-0.01, 0.05] | .05 |  |  |  |  |
| Satisfaction → General daydreaming | 0.01 [-0.03, 0.04] | .02 |  |  |  |  |
| Partner-related daydreaming → General daydreaming | 0.06 [-0.07, 0.18] | .05 |  |  |  |  |
| General daydreaming → Partner-related daydreaming | 0.05 [-0.03, 0.13] | .06 |  |  |  |  |
| First-order autoregressive effects |  |  |  |  |  |  |
| Partner-related daydreaming | 0.45^***^ [ 0.33, 0.57] | .48 |  |  |  |  |
| General daydreaming | 0.53^***^ [ 0.42, 0.64] | .53 |  |  |  |  |
| Satisfaction | 0.58^***^ [ 0.49, 0.68] | .61 |  |  |  |  |
| Second-order autoregressive effects |  |  |  |  |  |  |
| Partner-related daydreaming | 0.22^***^ [ 0.11, 0.32] | .25 |  |  |  |  |
| General daydreaming | 0.24^***^ [ 0.12, 0.37] | .25 |  |  |  |  |
| Satisfaction | 0.28^***^ [ 0.15, 0.40] | .30 |  |  |  |  |
| Concurrent correlations | Time 1  *r* [95% CI] |  |  | Time 2  *r* [95% CI] |  | Time 3  *r* [95% CI] |
| Partner-related daydreaming ↔ Satisfaction | .19^***^ [.08, .29] |  |  | .03 [-.10, .16] |  | .15 [-.01, .31] |
| General daydreaming ↔ Satisfaction | .03 [-.07, .14] |  |  | -.05 [-.19, .10] |  | .02 [-.14, .18] |
| Partner-related daydreaming ↔ General daydreaming | .68^***^ [.59, .76] |  |  | .55^***^ [.41, .70] |  | .65^***^ [.54, .75] |
| Investment (Model fit: χ^2^(15) = 21.99, *p* = .108, CFI = .993, SRMR = .034, RMSEA = .038 90% CI [.000, .069]) | | | | | | |
| Cross-lagged effects | *B* [95% CI] | β |  |  |  |  |
| Partner-related daydreaming → Investment | 0.33^**^ [ 0.14, 0.52] | .18 |  |  |  |  |
| General daydreaming → Investment | -0.21^**^ [-0.37, -0.05] | -.13 |  |  |  |  |
| Investment → Partner-related daydreaming | 0.02 [-0.02, 0.06] | .04 |  |  |  |  |
| Investment → General daydreaming | 0.01 [-0.03, 0.06] | .02 |  |  |  |  |
| Partner-related daydreaming → General daydreaming | 0.05 [-0.08, 0.18] | .05 |  |  |  |  |
| General daydreaming → Partner-related daydreaming | 0.04 [-0.04, 0.12] | .05 |  |  |  |  |
| First-order autoregressive effects |  |  |  |  |  |  |
| Partner-related daydreaming | 0.45^***^ [ 0.33, 0.57] | .48 |  |  |  |  |
| General daydreaming | 0.52^***^ [ 0.41, 0.64] | .53 |  |  |  |  |
| Investment | 0.45^***^ [ 0.34, 0.56] | .46 |  |  |  |  |
| Second-order autoregressive effects |  |  |  |  |  |  |
| Partner-related daydreaming | 0.22^***^ [ 0.12, 0.33] | .26 |  |  |  |  |
| General daydreaming | 0.24^***^ [ 0.12, 0.37] | .25 |  |  |  |  |
| Investment | 0.30^***^ [ 0.17, 0.43] | .31 |  |  |  |  |
| Concurrent correlations | Time 1  *r* [95% CI] |  |  | Time 2  *r* [95% CI] |  | Time 3  *r* [95% CI] |
| Partner-related daydreaming ↔ Investment | .28^***^ [.18, .38] |  |  | .18^***^ [.06, .30] |  | .12 [-.02, .26] |
| General daydreaming ↔ Investment | .10 [-.01, .22] |  |  | .10 [-.05, .24] |  | .04 [-.10, .19] |
| Partner-related daydreaming ↔ General daydreaming | .68^***^ [.59, .76] |  |  | .55^***^ [.41, .69] |  | .65^***^ [.55, .75] |
| Alternatives (Model fit: χ^2^(15) = 15.48, *p* = .418, CFI = .999, SRMR = .028, RMSEA = .010 90% CI [.000, .054]) | | | | | | |
| Cross-lagged effects | *B* [95% CI] | β |  |  |  |  |
| Partner-related daydreaming → Alternatives | 0.01 [-0.16, 0.18] | .01 |  |  |  |  |
| General daydreaming → Alternatives | 0.07 [-0.06, 0.21] | .05 |  |  |  |  |
| Alternatives → Partner-related daydreaming | -0.03 [-0.07, 0.01] | -.05 |  |  |  |  |
| Alternatives → General daydreaming | 0.03 [-0.02, 0.07] | .04 |  |  |  |  |
| Partner-related daydreaming → General daydreaming | 0.07 [-0.06, 0.20] | .06 |  |  |  |  |
| General daydreaming → Partner-related daydreaming | 0.06 [-0.03, 0.14] | .07 |  |  |  |  |
| First-order autoregressive effects |  |  |  |  |  |  |
| Partner-related daydreaming | 0.45^***^ [ 0.33, 0.57] | .48 |  |  |  |  |
| General daydreaming | 0.52^***^ [ 0.40, 0.63] | .52 |  |  |  |  |
| Alternatives | 0.44^***^ [ 0.34, 0.54] | .45 |  |  |  |  |
| Second-order autoregressive effects |  |  |  |  |  |  |
| Partner-related daydreaming | 0.22^***^ [ 0.12, 0.33] | .26 |  |  |  |  |
| General daydreaming | 0.24^***^ [ 0.12, 0.36] | .24 |  |  |  |  |
| Alternatives | 0.27^***^ [ 0.13, 0.41] | .28 |  |  |  |  |
| Concurrent correlations | Time 1  *r* [95% CI] |  |  | Time 2  *r* [95% CI] |  | Time 3  *r* [95% CI] |
| Partner-related daydreaming ↔ Alternatives | -.01 [-.12, .11] |  |  | -.03 [-.16, .09] |  | .10 [-.04, .24] |
| General daydreaming ↔ Alternatives | .18^**^ [.08, .28] |  |  | .06 [-.07, .19] |  | .21^**^ [.06, .36] |
| Partner-related daydreaming ↔ General daydreaming | .68^***^ [.59, .76] |  |  | .56^***^ [.42, .70] |  | .65^***^ [.55, .75] |
| *Note*. *B* = unstandardized coefficient. CI = confidence interval. β = average standardized coefficient. *r* = correlation coefficient. Coefficients from control variables to each variable were omitted for clarity of the presentation.  ^*^*p* < .05. ^**^*p* < .01. ^***^*p* < .001 | | | | | | |

**Supplemental Material 3: Details of cluster analysis on attachment style.**

To determine the number of clusters required to specify different types of attachment styles, we first conducted a hierarchical cluster analysis. Clustering validity indices indicated that the best number of clusters was three. Moreover, the dendrogram suggests that the three clusters would be good for categorizing participants (Figure S1). Therefore, we conducted a k-means non-hierarchical cluster analysis, setting three clusters and centroids identified by hierarchical cluster analysis to form the final clusters. As shown in Figure S2, the results clearly revealed the three attachment styles proposed by Hazan and Shaver (1987): secure (low anxiety and low avoidance; *n* = 118), anxious (high anxiety and middle avoidance; *n* = 76), and avoidant (middle anxiety and high avoidance; *n* = 133).

**Figure S1**

*Dendrogram from the Hierarchical Cluster Analysis*


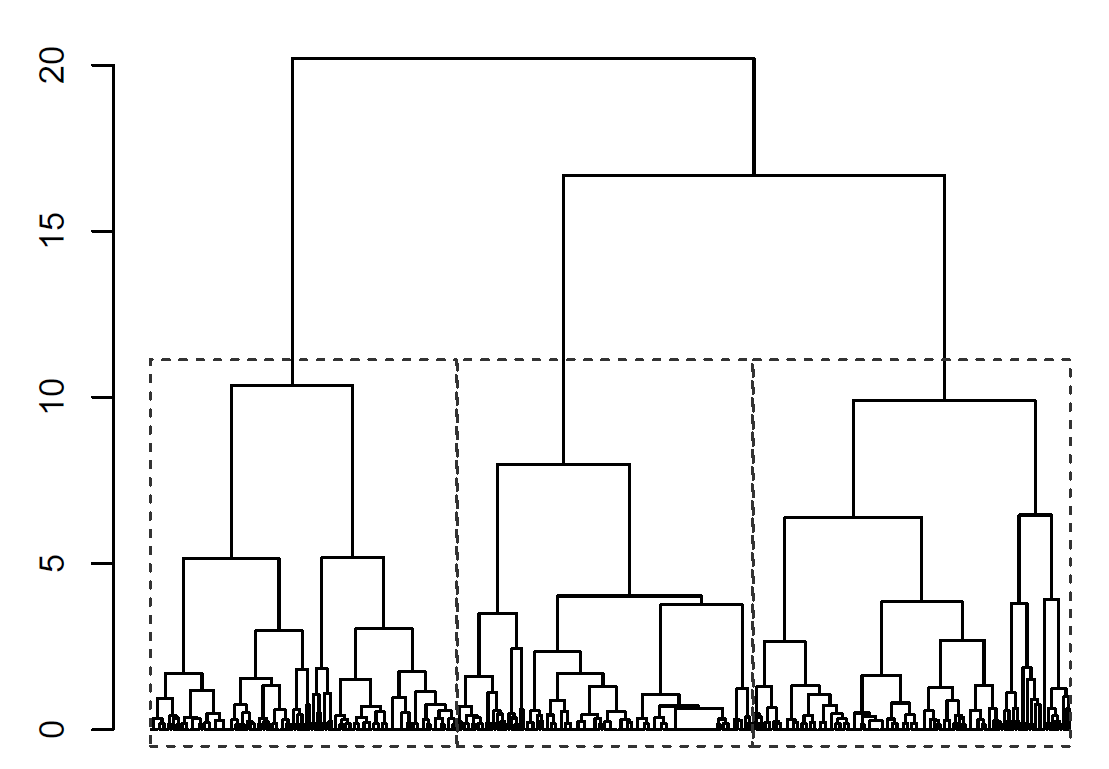


**Figure S2**

*
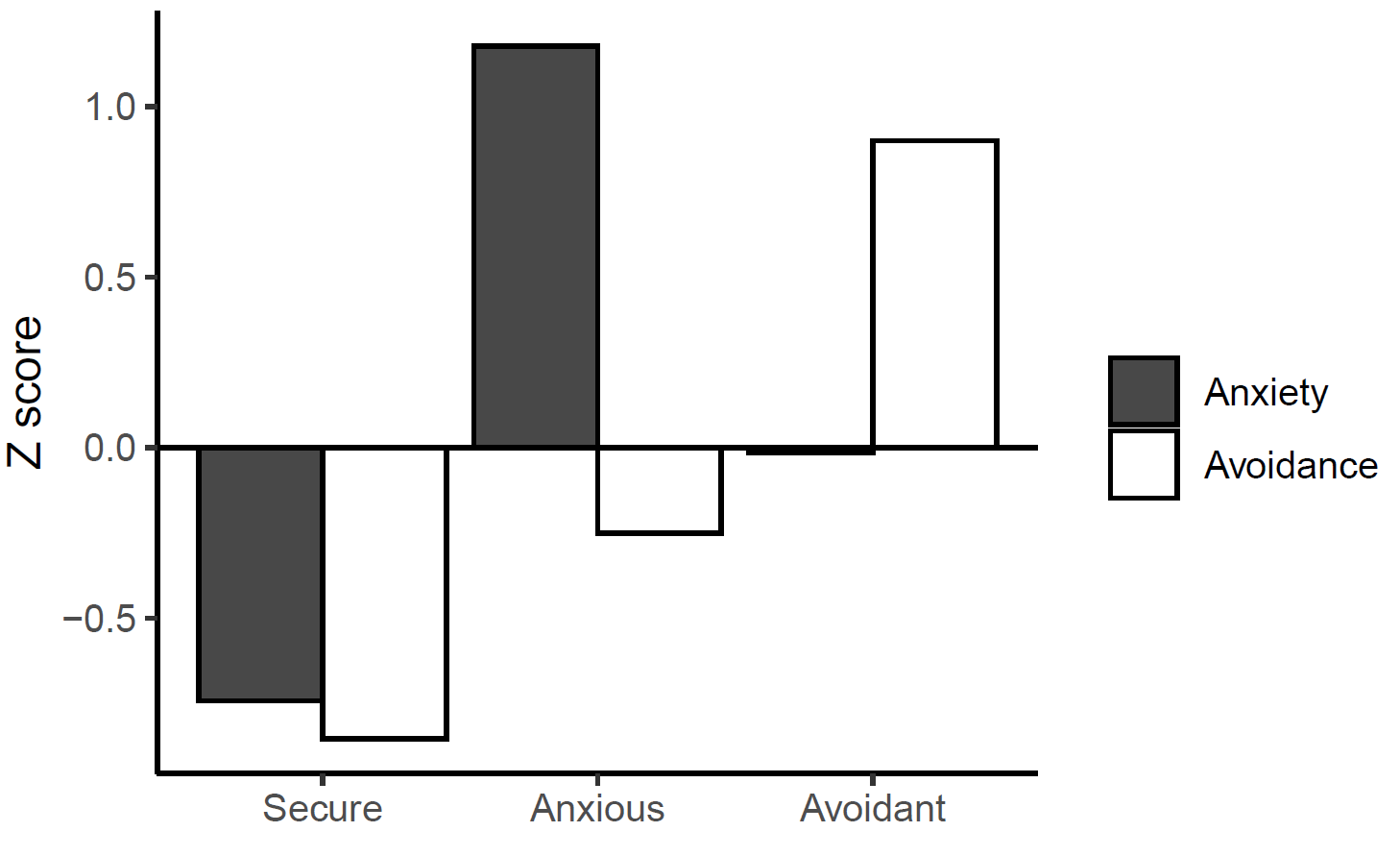
Z-scores of Anxiety and Avoidance for Three Attachment Style Clusters from the k-means Cluster Analysis*

Table S1 summarizes the results of the mean comparison between attachment styles for each time variable. Univariate analysis of variance (ANOVA) revealed that partner-related daydreaming was significantly different across attachment styles. Post hoc analyses showed that it was highest for the anxious group, followed by the secure and avoidant groups. Contrastingly, the difference in general daydreaming was weaker; although it was significantly higher for the anxious group than for the avoidant group, there were no significant differences between the secure group and the remaining two groups.

Commitment and satisfaction were significantly higher for the secure group, followed by the anxious and avoidant groups. Investment was significantly higher in the secure and anxious groups than in the avoidant group. Alternatives was lower for the secure group than for the anxious and avoidant groups. Marital duration was the longest in the avoidant group, followed by the secure and anxious groups. There were no significant gender differences in the attachment style types. Overall, the results were consistent with attachment style theory (Hazan & Shaver, 1987).

**Table S1**

*Mean Comparisons between Attachment Styles for Each Variable*

|  | Secure |  | Anxious |  | Avoidant |  |  |  |
| --- | --- | --- | --- | --- | --- | --- | --- | --- |
| Variable | *M* (*SD*) |  | *M* (*SD*) |  | *M* (*SD*) |  | *F* | *η^2^* [95% CI] |
| Time 1 |  |  |  |  |  |  |  |  |
| Gender^a^ | 0.61 (0.49) |  | 0.46 (0.50) |  | 0.48 (0.50) |  | 2.88 | .02 [.00, .05] |
| Marital duration | 13.89_b_ (12.88) |  | 11.54_b_ (11.09) |  | 18.87_a_ (12.81) |  | 9.68^***^ | .06 [.02, .11] |
| Partner-related daydreaming | 1.86_b_ (0.72) |  | 2.06_a_ (0.70) |  | 1.62_c_ (0.61) |  | 10.88^***^ | .06 [.02, .11] |
| General daydreaming | 2.16_ab_ (0.80) |  | 2.36_a_ (0.70) |  | 2.00_b_ (0.72) |  | 5.54^**^ | .03 [.00, .08] |
| Commitment | 6.29_a_ (0.80) |  | 5.85_b_ (0.97) |  | 4.70_c_ (1.33) |  | 71.56^***^ | .31 [.23, .38] |
| Satisfaction | 5.83_a_ (1.06) |  | 5.21_b_ (1.37) |  | 4.08_c_ (1.42) |  | 58.87^***^ | .27 [.19, .34] |
| Investment | 4.56_a_ (1.19) |  | 4.66_a_ (1.00) |  | 3.47_b_ (1.07) |  | 41.73^***^ | .20 [.13, .28] |
| Alternatives | 3.44_b_ (1.13) |  | 3.83_a_ (1.04) |  | 3.96_a_ (0.95) |  | 8.30^***^ | .05 [.01, .10] |
| Time 2 |  |  |  |  |  |  |  |  |
| Partner-related daydreaming | 1.67_a_ (0.68) |  | 1.75_a_ (0.53) |  | 1.52_a_ (0.55) |  | 3.18^*^ | .02 [.00, .07] |
| General daydreaming | 1.99 (0.78) |  | 2.18 (0.62) |  | 1.89 (0.74) |  | 2.87 | .02 [.00, .07] |
| Commitment | 6.03_a_ (0.91) |  | 5.47_b_ (1.13) |  | 4.55_c_ (1.26) |  | 43.55^***^ | .26 [.17, .34] |
| Satisfaction | 5.57_a_ (1.08) |  | 5.03_b_ (1.28) |  | 4.01_c_ (1.43) |  | 37.09^***^ | .23 [.14, .31] |
| Investment | 4.46_a_ (1.13) |  | 4.33_a_ (1.03) |  | 3.43_b_ (1.13) |  | 24.23^***^ | .16 [.09, .24] |
| Alternatives | 3.43_b_ (1.02) |  | 3.94_a_ (0.89) |  | 3.90_a_ (1.04) |  | 6.67^**^ | .05 [.01, .11] |
| Time 3 |  |  |  |  |  |  |  |  |
| Partner-related daydreaming | 1.62_b_ (0.68) |  | 1.98_a_ (0.53) |  | 1.55_b_ (0.55) |  | 8.58^***^ | .07 [.02, .15] |
| General daydreaming | 1.86_b_ (0.71) |  | 2.34_a_ (0.72) |  | 1.89_b_ (0.73) |  | 7.77^***^ | .07 [.01, .14] |
| Commitment | 6.06_a_ (0.96) |  | 5.33_b_ (1.02) |  | 4.67_c_ (1.18) |  | 35.37^***^ | .25 [.15, .34] |
| Satisfaction | 5.64_a_ (1.06) |  | 4.81_b_ (1.10) |  | 3.97_c_ (1.35) |  | 40.35^***^ | .27 [.18, .36] |
| Investment | 4.37_a_ (1.09) |  | 4.37_a_ (0.92) |  | 3.45_b_ (1.13) |  | 19.62^***^ | .15 [.07, .24] |
| Alternatives | 3.40_b_ (1.04) |  | 3.95_a_ (0.90) |  | 3.77_a_ (0.98) |  | 5.28^**^ | .05 [.00, .11] |
| *Note*. Means with different subscripts differ at the adjusted *p* = .05 level by Holm’s method.  ^a^ Male = 0, Female = 1. The χ^2^ test was also non-significant (χ^2^ (2) = 5.71, *p* = .057).  ^*^*p* < .05. ^**^*p* < .01. ^***^*p* < .001 | | | | | | | | |

**References**

Gordon, A. M., Impett, E. A., Kogan, A., Oveis, C., & Keltner, D. (2012). To have and to hold: Gratitude promotes relationship maintenance in intimate bonds. *Journal of Personality and Social Psychology*, *103*(2), 257–274. <https://doi.org/10.1037/a0028723>

John, O. P., Donahue, E. M., & Kentle, R. L. (1991). *The Big Five Inventory—Versions 4a and 54.*

McCullough, M. E., Emmons, R. A., & Tsang, J. A. (2002). The grateful disposition: A conceptual and empirical topography. *Journal of Personality and Social Psychology*, *82*(1), 112–127. <https://doi.org/10.1037//0022-3514.82.1.112>

Roets, A., & Van Hiel, A. (2011). Item selection and validation of a brief, 15-item version of the Need for Closure Scale. *Personality and Individual Differences*, *50*(1), 90–94. <https://doi.org/10.1016/j.paid.2010.09.004>

Scheier, M. F., Carver, C. S., & Bridges, M. W. (1994). Distinguishing optimism from neuroticism (and trait anxiety, self-Mastery, and self-Esteem): A reevaluation of the Life Orientation Test. *Journal of Personality and Social Psychology*, *67*(6), 1063–1078. <https://doi.org/10.1037/0022-3514.67.6.1063>
